# Understanding contact gating in Schottky barrier transistors from 2D channels

**DOI:** 10.1038/s41598-017-12816-3

**Published:** 2017-10-03

**Authors:** Abhijith Prakash, Hesameddin Ilatikhameneh, Peng Wu, Joerg Appenzeller

**Affiliations:** 1 School of Electrical and Computer Engineering, Purdue University, West Lafayette, 47907 Indiana USA; 2 Birck Nanotechnology Center, Purdue University, West Lafayette, 47907 Indiana USA; 3 Network for Computational Nanotechnology, 207 S. Martin Jischke Drive, West Lafayette, 47907 Indiana USA

## Abstract

In this article, a novel two-path model is proposed to quantitatively explain sub-threshold characteristics of back-gated Schottky barrier FETs (SB-FETs) from 2D channel materials. The model integrates the “conventional” model for SB-FETs with the phenomenon of contact gating – an effect that significantly affects the carrier injection from the source electrode in back-gated field effect transistors. The two-path model is validated by a careful comparison with experimental characteristics obtained from a large number of back-gated WSe_2_ devices with various channel thicknesses. Our findings are believed to be of critical importance for the quantitative analysis of many three-terminal devices with ultrathin body channels.

## Introduction

Over the years, fabrication of back-gated (BG-) field-effect transistors (FETs) has become the most common way to build a three-terminal device on emerging materials to investigate their intrinsic properties and to understand the resulting carrier transport^1–17^. BG-FETs have been an attractive option particularly due to the ease of device fabrication and the resulting high yield. While often not employing a scaled dielectric, there have been numerous instances where a back-gating approach has been utilized for the initial demonstration of novel phenomena such as band-to-band tunneling, the impact of strain or observation of quantum oscillations in 2D systems, to just name a few^18–27^. What makes back-gated device structures special is that different from a conventional device layout, the entire channel segment underneath the source/drain contact region is under some influence of the gate. It is this particular behavior that needs to be understood in order for any quantitative device analysis to be relevant, which is the topic of this article.

Since chemical doping of low-dimensional materials is challenging and is still in its infancies, a transistor structure with highly doped source and drain regions connected to a gated channel, as employed for conventional metal-oxide semiconductor (MOS) FETs, is not common for exploratory devices. In fact, source and drain metal contacts are typically directly deposited onto the novel channel material, in this way only making direct contact to the very top. Such a structure when gated is commonly referred to as Schottky barrier (SB)-FET. Frequently, this top-contact design is combined with the use of a heavily doped substrate (*e.g*. silicon) isolated from the channel through a dielectric (*e.g*. silicon dioxide) as a large area gate of the device test structure, thus bringing the entire channel, including the source-to-channel and the drain-to-channel region under the gate control. Analyzing this type of structure has been the focus of many research articles and the description of SB-FETs in terms of a gated channel that is connected to a fixed barrier at the metal-to-channel interface (the Schottky barrier) has been successfully employed for a number of model systems including 1D channels like Si nanowires, carbon nanotubes and 2D channels like black phosphorus, MoS_2_ and alike^28–32^.

In this article, we will discuss in how far the “conventional” Schottky barrier model^31,32^ needs to be extended in general to include contact gating, an effect that had been discussed by us in 2009 in the context of graphene devices^33^, to accurately describe the sub-threshold device characteristics from most two-dimensional (2D) materials. In particular, we propose here a general, physics-based parameter-free model to describe the electrical characteristics of back-gated SB-FETs with 2D channels, and demonstrate its validity by employing it to successfully explain the experimentally obtained characteristics of back-gated WSe_2_ SB-FETs for various channel thicknesses.

## Results and Discussion

Any current I_D_ in an SB-FET can be associated with either: (i) thermal current from purely thermionic carrier injection over the Schottky barrier or (ii) Schottky barrier current due to thermally assisted tunneling of charge carriers through the Schottky barrier. The conventional SB-FET model describes the sub-threshold region (OFF state) of the transfer characteristic (I_D_-V_GS_) with the help of a single equation, using Landauer formalism, assuming that the gate’s control only extends over the channel (*i.e*. without including the segments underneath the source and drain contacts). As per the conventional SB-FET model^31,32^, the source-injected electron current per unit channel width is given by

1 $${{\rm{I}}}_{{\rm{D}}}=\frac{2{\rm{q}}}{{\rm{h}}}{\int }_{{{\rm{E}}}_{{\rm{C}}}}^{\infty }{\rm{M}}({\rm{E}}){\rm{T}}({\rm{E}}){\rm{f}}({\rm{E}}){\rm{dE}}$$

where M(E) is the number of modes per unit width given by

2 $${\rm{M}}({\rm{E}})=\frac{2}{{\rm{h}}}\sqrt{2{{\rm{m}}}_{{\rm{e}}}({\rm{E}}-{{\rm{E}}}_{{\rm{C}}})}$$

For E < Φ_n_, T(E) is the probability of transmission through the Schottky contact as calculated by WKB approximation and is given by

3 $${\rm{T}}({\rm{E}})=\exp (-\frac{8{\rm{\pi }}}{{\rm{3h}}}\sqrt{2{{\rm{m}}}_{{\rm{e}}}{({{\rm{\Phi }}}_{{\rm{n}}}-{\rm{E}})}^{3}}\frac{{\rm{\lambda }}}{{{\rm{\Phi }}}_{{\rm{n}}}\,-\,{{\rm{E}}}_{{\rm{C}}}})$$

For energies greater than the Schottky barrier height for electrons (Φ_n_), the probability of transmission is unity as this corresponds to pure thermal injection.

**Figure 1:**
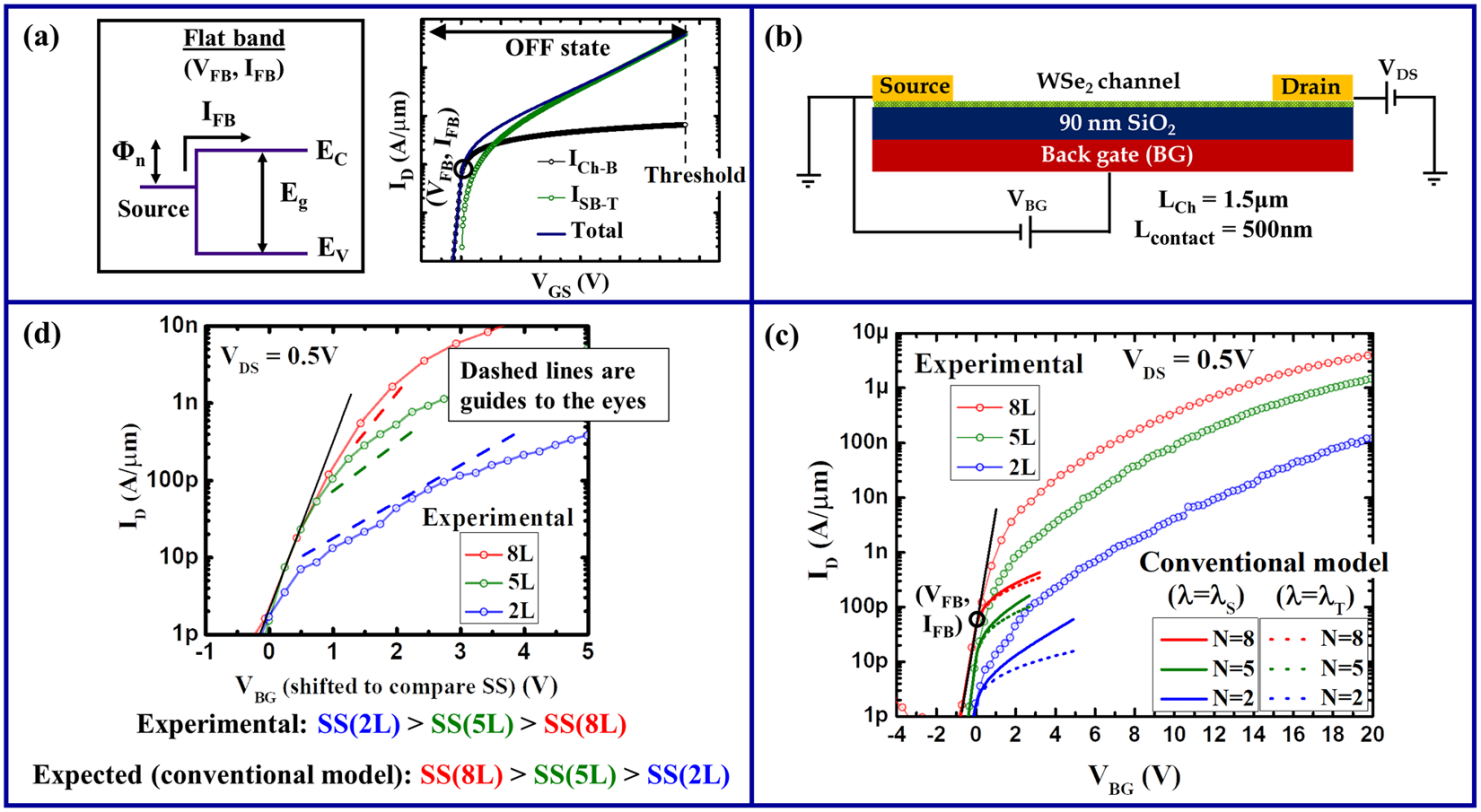
(**a**) Various components of current in the conventional SB-FET model. (**b**) Schematics of a back-gated WSe_2_ Schottky barrier FET. (**c**) Comparison of the experimental device characteristics with simulations based on the conventional SB-FET model. (**d**) Comparison of SS for the same set of experimental transfer characteristics as in (**c**).

In the above equations, E is the electron energy with respect to the metal Fermi level at the source, f(E) is the Fermi function at the source given by

$${\rm{f}}({\rm{E}})={[1+\exp (\frac{{\rm{E}}}{{{\rm{k}}}_{{\rm{B}}}{\rm{T}}})]}^{-1},$$

m_e_ is the effective tunneling mass for electrons which is usually expressed as a multiple of the free-electron mass m_0_, and E_C_ is the gate-bias controlled conduction band minimum in the channel. The gate voltage at which E_C_ = Φ_n_ is known as the flat-band voltage (V_FB_), which separates the thermal injection dominated gate voltage range from the Schottky barrier dominated one. In fact, I_D_ can be divided into two components I_Ch-B_ and I_SB-T_ (*i.e*., I_D_ = I_Ch-B_ + I_SB-T_) where I_Ch-B_ is due to thermal injection, limited by the channel potential below flat-band and by the Schottky barrier above flat-band. I_SB-T_ is the additional current injected by tunneling through the Schottky barrier above flat-band (Fig. 1(a)). Since I_Ch-B_ flows through the channel even if there is no tunneling through the Schottky barrier, it is regarded as the basic channel current.

λ is the characteristic length scale which defines the distance over which the potential changes from the metal-semiconductor interface to the channel. Several equations have been proposed in the literature for λ in an ultrathin-body channel^34–36^, the two prominent ones being: (i) a square root scaling length given by

$${{\rm{\lambda }}}_{{\rm{S}}}=\sqrt{\frac{{{\rm{\varepsilon }}}_{\mathrm{body}-{\rm{x}}}}{{{\rm{\varepsilon }}}_{\mathrm{ox}}}{{\rm{t}}}_{\mathrm{body}}{{\rm{t}}}_{\mathrm{ox}}}$$

and (ii) a generalized scaling length λ_T_, the value of which is obtained by solving the equation

$$\frac{1}{{{\rm{\varepsilon }}}_{{\rm{ox}}}}\,\tan \,[\frac{2{{\rm{t}}}_{{\rm{ox}}}}{{{\rm{\lambda }}}_{{\rm{T}}}}]+\frac{1}{{{\rm{\varepsilon }}}_{{\rm{body}}-{\rm{x}}}}\,\tan \,[\frac{2{{\rm{t}}}_{{\rm{body}}}}{{{\rm{\lambda }}}_{{\rm{T}}}}]=0.$$

In the above expressions, t_ox_ is the thickness of gate oxide, ε_ox_ denotes the dielectric constant of the gate oxide, ε_body-x_ refers to the in-plane dielectric constant of the channel material and t_body_ is the body thickness of the ultrathin channel.

If the band movement in the channel is not controlled by the gate voltage (V_GS_) in a one-to-one fashion, the entire I_D_-V_GS_ curve resulting from the conventional SB-FET model is “stretched” along the V_GS_-axis by a factor γ (band movement factor) which is the ratio of the change in gate voltage to the change in actual channel potential, thereby deteriorating the inverse sub-threshold slope (SS = d(V_GS_)/d(log(I_D_))) for both, the thermal and the SB dominated part of the characteristics. This implies that in the case of thermal injection dominated currents, SS would deviate from its ideal value of 60 mV/dec at room temperature, becoming 60 γmV/dec and in the case of Schottky barrier currents, SS, which is always larger than 60 mV/dec^29,30,37–39^, will further increase by the same factor γ.

### Necessity of a new model

To test the validity of a model, benchmarking with experimental results is necessary. For such a comparison in the case of back-gated Schottky barrier transistors with 2D channels, a 2D material which exhibits a prominent Schottky barrier current branch as well as a thermal branch observable above the measurement noise floor, needs to be chosen. WSe_2_, which is an important member of the family of two-dimensional transition metal dichalcogenides (TMDs)^18,24,40–45^ is known to satisfy these requirements^18,46^.

In order to fabricate back-gated WSe_2_ SB-FETs, flakes of WSe_2_ were micro-mechanically exfoliated on top of substrates with 90 nm SiO_2_ thermally grown on highly doped silicon. Flakes of various thicknesses were identified by means of optical contrast after proper calibration and atomic force microscopy (AFM) in tapping mode. Electron beam lithography followed by electron beam evaporation was used to define source and drain contacts, each designed to have a contact length (L_contact_) of 500 nm. Ni was used as the contact metal. The channel lengths for all the devices were designed to be 1.5 μm and the highly doped Si was used as the back-gate electrode. A schematic of the device structure is shown in Fig. 1(b). All electrical measurements were carried out at room-temperature at a vacuum of ~10^−6^ Torr in a Lake Shore probe station using an Agilent semiconductor parameter analyzer.

For each device (except the ones with single layer channels) the flat-band voltage (V_FB_) was determined by carefully identifying the point of deviation from the thermal branch which is the point where I_D_ deviates from its exponential dependence on V_BG_ in the lowest current range (see Fig. 1(c)). From the corresponding current I_FB_, the Schottky barrier height Φ_n_ was extracted using the equation

$${{\rm{I}}}_{{\rm{FB}}}=\frac{2{\rm{q}}}{{\rm{h}}}{\int }_{{{\rm{\Phi }}}_{{\rm{n}}}}^{\infty }{\rm{M}}({\rm{E}}){\rm{f}}({\rm{E}}){\rm{dE}},$$

which is nothing but equation (1) at flat-band, by using an electron effective mass of 0.36m_0_, a value that is in accord with what has been reported in the literature^44,47,48^. All Schottky barrier heights extracted in this way ranged between 0.4 eV to 0.5 eV, depending on the body thickness as will be further discussed later. Since all measurements were performed at a drain bias of 0.5 V which is greater than the Schottky barrier height, the drain side Schottky contact impact is eliminated^1,32^.

The value of ${\rm{\gamma }}$ for each device was determined experimentally by comparing the inverse sub-threshold slope (SS) of its thermal branch with 60 γmV/dec. Channel thickness dependent values of the dielectric constant were obtained with the help of values reported in the literature^49^ (see supplementary information I) by assuming t_body_ to be 0.7 nm times the number of WSe_2_ layers (N) in the channel^42,50^.

Utilizing the extracted Schottky barrier heights from above, we employed the conventional SB-FET model, with both expressions - λ_S_ and λ_T_ - for λ, to explain our experimental results. Figure 1(c) illustrates the discrepancy between experimental data and the simulations. Not only does the thermal current transition at V_FB_ into a Schottky barrier dominated current that is too low, but more importantly the gate voltage at which the conventional model predicts the device characteristics to transition into their ON-state (the V_BG_-values at which the simulated curves end) is not even remotely close to where currents start to flatten out in the experimental curves which is for V_BG_ ~ 15 V to 30 V. Attempts to artificially adjust parameters to achieve a better match between the conventionally modeled electrical response and the experimental data in terms of current levels requires much smaller Schottky barrier heights than those extracted from the flat band currents. However, these values are unrealistic considering the ambipolar nature of the experimental transfer characteristics (see supplementary information II) combined with the values of bandgaps previously extracted by us^37^. Moreover, even artificially correcting the current levels does still not yield an overall better fit (see supplementary information III in this context). Similarly, artificially varying ε_body-x_ to its minimum possible value was also explored to achieve a fit with the conventional SB-FET model, but without any success. One of the most important discrepancies can be seen in Fig. 1(d), which shows that in addition to the other above arguments the experimental trend in SS with respect to body thickness and ε_body-x_ is opposite to that predicted by the conventional model. A smaller body thickness should decrease the scaling length through both, a decrease in t_body_ and a decrease in ε_body-x_ (see Figure S1). All of the above implies that a major aspect in the description of the behavior of back-gated WSe_2_ Schottky barrier FETs is missing in the conventional SB model.

### Importance of gate geometry

**Figure 2:**
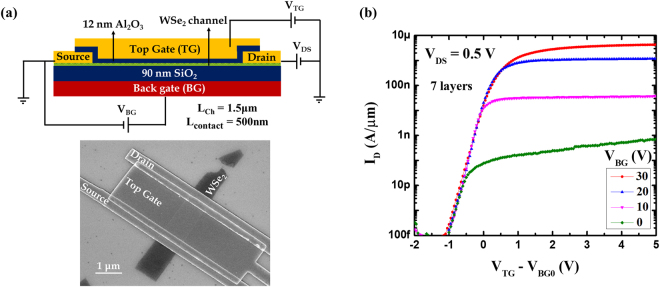
(**a**) Modified device structure after the fabrication of a top gate along with the corresponding SEM image. (**b**) Top-gated transfer characteristics of a representative device for different values of V_BG_ after compensating for the back gate induced threshold shifts V_BG0_.

The failure of the conventional SB-FET model in the domain of back gated 2D transistors, considering its success in modeling top gated transistors on 2D channels such as ultrathin body Si^51^, brings up the question: “Is there a fundamental difference between these two structures?” Since the conventional SB-FET model treats a top gate and a back gate identically, comparing top and bottom gated devices allows identifying their different impact on the channel. For that, we fabricated top gates on previously characterized back-gated devices covering the entire channel region in-between the source and drain contacts with 12 nm thick Al_2_O_3_ using atomic layer deposition (ALD) and employing electron beam lithography plus electron beam evaporation to fabricate the top gates. Ni was used as the top gate metal. The resulting device structure, along with the corresponding SEM image, is shown in Fig. 2(a) and device characteristics for several V_BG_ conditions while sweeping the top gate voltage V_TG_ are displayed in Fig. 2(b).

If the two gates’ impact on the channel is identical, changing the fixed voltage applied to one gate should result only in a threshold voltage shift in the transfer characteristics when the voltage applied to the other gate is swept. This is clearly not the case in Fig. 2(b) where the achievable ON-state current is a strong function of V_BG_, which implies that carrier injection is ultimately limited by the back gate. This observation is in accord with the experimental results reported by H.C.P. Movva, *et al*.^52^, considering that the top gate and back gate are reversed in their device structure. As it is evident from Fig. 2(b), the top gate can only turn the device OFF, *i.e*., it can only block the current. It can however not increase the current beyond a certain point by itself. This implies that the back gate can impact the channel region in portions not accessible to the top gate, which are the TMD segments right underneath the source and drain contacts. Since the back gate impact is substantial enough to modify the ON-state current levels by orders of magnitude, the conventional Schottky barrier model requires including these particular regions in the calculations of device characteristics explicitly which is the topic of the next section.

### A new two-path model for back-gated Schottky barrier field-effect transistors

**Figure 3:**
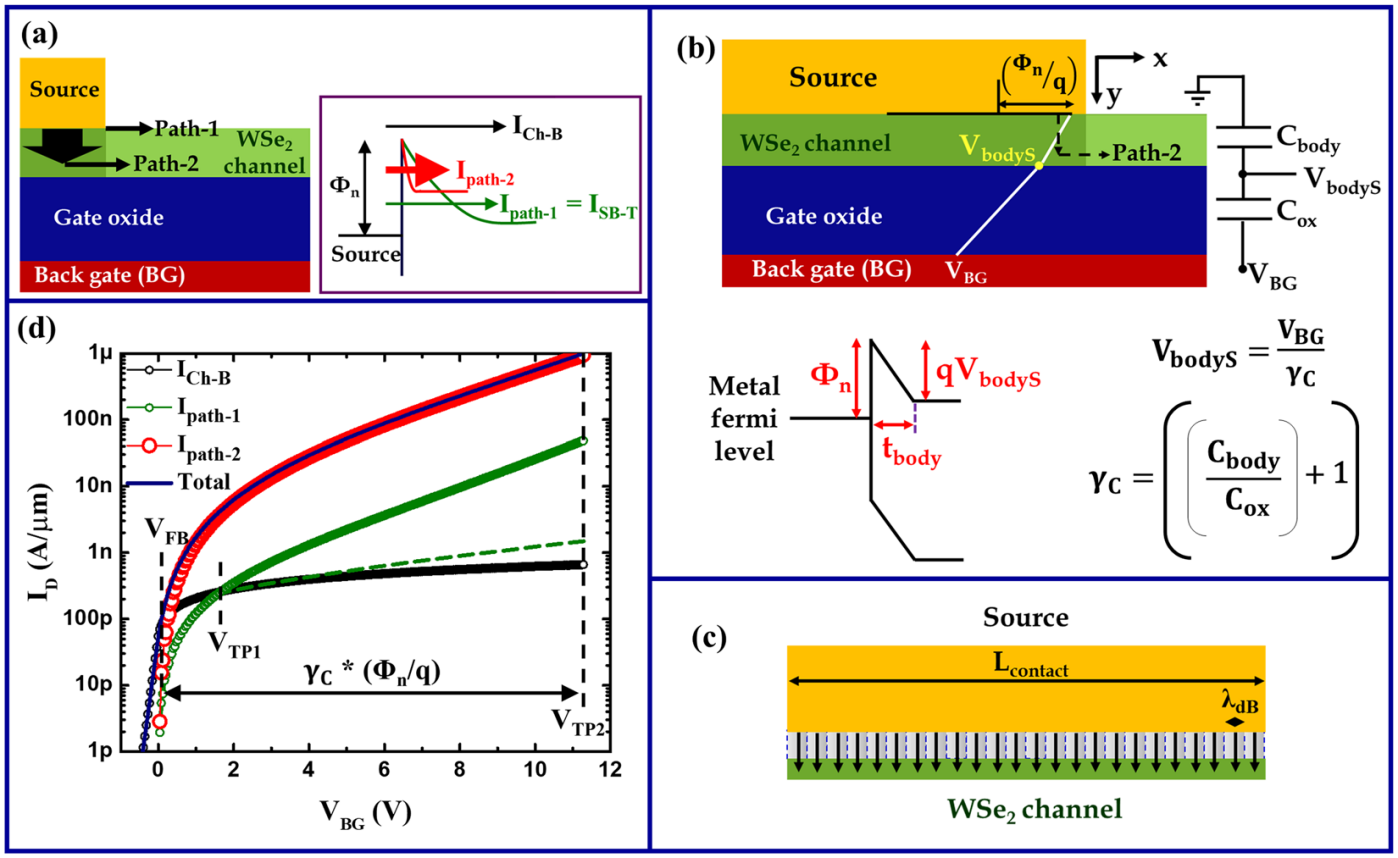
Illustration of the two-path model for back-gated SB-FETs where (**a**) shows the two injection paths, (**b**) explains diagrammatically the injection via path-2 and (**c**) presents a pictorial representation of the number of injecting states N_i_ along the contact length. Shown in (**d**) is a typical transfer characteristic of a back-gated WSe_2_ SB-FET, along with the individual contributions of each of the two paths, calculated as per the new model for a Schottky barrier height of 0.4 eV and a body thickness of 7 nm by assuming a square root scaling length λ_S_ for path-1. Green circles assume continuous band movement for path-1 even above its threshold (V_TP1_) whereas green dashed lines assume slowed down band movement for path-1 above threshold as described in the text.

In order to account for the aforementioned “additional” effect of a back gate in the contact region, we are proposing here a so called “two-path” model (see Fig. 3(a)). In this model, similar to the conventional model, the total current below flat-band is limited to the basic channel current I_Ch-B_ since the channel resistance, by virtue of its barrier height, dominates the total resistance in this regime. Beyond flat-band, apart from allowing I_Ch-B_, the back gate has two separate functions: (i) The back gate modulates the carrier injection via Schottky barrier tunneling right at the edge of the source-to-channel region as in the conventional SB-model (path-1) and (ii) allows simultaneously for injection into deep-lying layers of the TMD flake due to the electric field that is built up by V_BG_ underneath the source contact (path-2). The sum of all these currents is the V_BG_-dependent total current through the entire device characteristics.

In order to model path-2 for carrier injection, it is important to examine the potential profile in the channel region underneath the contacts in a back-gated device. Since the gate voltage drops across two dielectrics, the semiconducting channel material and the back oxide, a simple capacitance divider, as shown in Fig. 3(b), that treats the portion of the device underneath the source as a series arrangement of two parallel plate capacitors C_body_ and C_ox_ can be employed. Here C_body_ = ε_body-y_/t_body_ and C_ox_ = ε_ox_/t_ox_, where ε_body-y_ and ε_ox_ are the respective permittivities of the channel material and the oxide in y (out-of-plane) direction, t_body_ and t_ox_ are the respective thicknesses of the channel body and the gate oxide. Since we are dealing with the device’s OFF-state, the carrier density in the channel underneath the source contact is small and hence the potential profile along the thickness of the channel (y-direction) is almost linear. The total potential drop V_bodyS_ across the channel body under the source, in the y-direction, can be obtained by solving the above-described capacitance network to be V_BG_/${{\rm{\gamma }}}_{{\rm{C}}}$ where ${{\rm{\gamma }}}_{{\rm{C}}}$ is the band movement factor underneath the contact given by:

4 $${{\rm{\gamma }}}_{{\rm{C}}}=((\frac{{{\rm{t}}}_{{\rm{ox}}}\,\ast \,{{\rm{\varepsilon }}}_{{\rm{body}}-{\rm{y}}}}{{{\rm{t}}}_{{\rm{body}}}\,\ast \,{{\rm{\varepsilon }}}_{{\rm{ox}}}})+1)$$

Figure 3(b) shows the potential profile along the semiconducting channel underneath the source contact. Carrier injection along path-2 depends on the vertical electric field V_bodyS_/t_body_. We model this current as a tunneling current through a triangular barrier with the barrier height being equal to the Schottky barrier height Φ_n_ and the tunneling distance given by the channel thickness t_body_ as shown in Fig. 3(b). Accordingly, the current per unit channel width for path-2 can be written as

5 $${{\rm{I}}}_{{\rm{path}}2}=\frac{2{\rm{q}}}{{\rm{h}}}{\int }_{{{\rm{E}}}_{{\rm{CS}}}}^{{{\rm{\Phi }}}_{{\rm{n}}}}{{\rm{N}}}_{{\rm{i}}}{({\rm{E}}){\rm{M}}}_{{\rm{S}}}{({\rm{E}}){\rm{T}}}_{\mathrm{WKB}-{\rm{S}}}({\rm{E}}){\rm{f}}({\rm{E}})\mathrm{dE}$$

where E_CS_ = Φ_n_ − qV_bodyS_, is the conduction band minimum at the bottom of the channel body under the source contact, M_S_(E) captures the number of 2D modes per unit width and T_WKB-S_(E) is the probability of transmission through the triangular barrier along path-2 in WKB approximation. M_S_(E) and T_WKB-S_(E) are given by equations (2) and (3) respectively when E_C_ is replaced by E_CS_, and λ is replaced by t_body_. f(E) is the Fermi function at the source and N_i_(E) is the number of injecting states along the contact length L_contact_, which is given by

6 $${{\rm{N}}}_{{\rm{i}}}({\rm{E}})=\frac{{{\rm{L}}}_{{\rm{contact}}}}{{\rm{h}}}\sqrt{2{{\rm{m}}}_{{\rm{e}}}({\rm{E}}-{{\rm{E}}}_{{\rm{CS}}})}$$

To obtain the above expression for N_i_(E), we have assumed that the potential drop across the channel body, which is responsible for the carrier injection, is identical over the entire contact area A_C_ (A_C_ = device width*L_contact_). To calculate the current per unit width at any energy, the number of 2D modes per unit width M_S_(E) has to be multiplied by the number of injecting states N_i_(E) along the contact length L_contact_ (see supplementary note in this context). Since each injecting state “occupies” a length segment equal to the de-Broglie wavelength of an electron in the semiconductor (Fig. 3(c)) *i.e*., $2{\rm{\pi }}/{\rm{k}}$ where k is the magnitude of the wave vector, the total number of injecting states N_i_ along L_contact_ is equal to $\frac{{{\rm{L}}}_{\mathrm{contact}}}{(2{\rm{\pi }}/{\rm{k}})}$, which results in the expression presented in equation (6) when a parabolic energy dispersion in the semiconductor is assumed. When the back-gate voltage V_BG_ is varied, V_bodyS_ changes as V_BG_/γ_C_, E_CS_ changes as Φ_n_ − qV_bodyS_. Then N_i_ is calculated for every E-E_CS_ as per equation (6), and used in equation (5) to obtain I_path2_.

Figure 3(d) shows simulated transfer characteristic of a back-gated WSe_2_ SB-FET, along with the individual contributions of both the injection paths, calculated for a Schottky barrier height of 0.4 eV and a body thickness of 7 nm by assuming a square root scaling length λ_S_ for path-1. V_TP1_ and V_TP2_ in the figure refer to the threshold voltages of path-1 and path-2 respectively, where “threshold voltage” refers to the voltage at which the conduction band edge in the corresponding path gets aligned with the source Fermi level. I_path-1_ shown in the figure was calculated by assuming that the band movement for path-1 continues one-to-one with V_BG_/${\rm{\gamma }}$ even above its threshold V_TP1_. The consequence of this assumption is that when V_BG_ = V_TP2_, the conduction band in the conventional channel would be ~1.8 eV below the valance band edge at the source metal-to-semiconductor contact interface. Since this is a highly unrealistic situation, we have shown by the dashed green line, the case where the band movement slows down after V_TP1_ is reached and moves such that the conduction band in the channel reaches the valance band edge at the source metal-to-semiconductor contact interface when V_BG_ = V_TP2_. Since in both the cases, the contribution of I_path-1_ to the total current is negligible, assumptions regarding the band movement for path-1 above V_TP1_ do not make a considerable difference under the circumstances considered here. While calculating I_path-1_ for channel potentials exceeding 0.5 V above V_FB_, though the impact of the drain side Schottky barrier has been considered, it was found to have a negligible impact for the large V_DS_-value of 0.5 V considered here. It is important to realize that there is a significant difference in the electrostatic gate control of the potentials underneath the contact and in the conventional channel. Under the contact, the ratio of C_body_ and C_ox_ determines the band movement factor γ_C_^53^, resulting in a body-thickness and material dependent gate control whereas in the conventional channel, ${\rm{\gamma }}$ and hence the gate control is body-thickness independent and much stronger. As a result, path-1 reaches its threshold voltage V_TP1_ at a much smaller gate bias compared to path-2 V_TP2_ as shown in the figure. Since currents above flat band due to path-2 are much larger than those due to path-1 in the present case (see supplementary section IV for a counter example), the threshold voltage visible in the full device characteristic is that of path-2 and the resulting stretch of the transfer characteristics is much larger compared to that due to path-1 (Fig. 3(d)). As the strengths of the back gate control (*i.e*., ratios of change in channel potential to change in gate voltage) are different for the two paths, we have considered here an undoped channel that ensures that the band bending situations for path-1 and path-2 coincide at flat-band. Different band offsets might result from doping - intentional or unintentional - or from the work function difference between the top and bottom gates in case of double gated structures.

**Figure 4:**
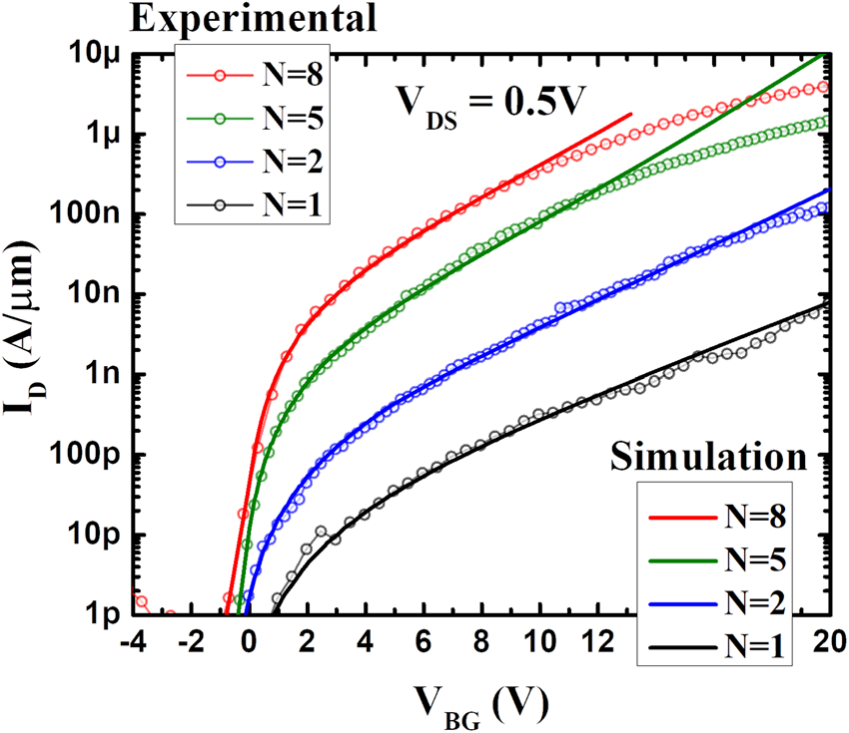
Comparison between the experimental device characteristics obtained from various back-gated WSe_2_ SB-FETs and simulations performed based on the new model.

**Figure 5:**
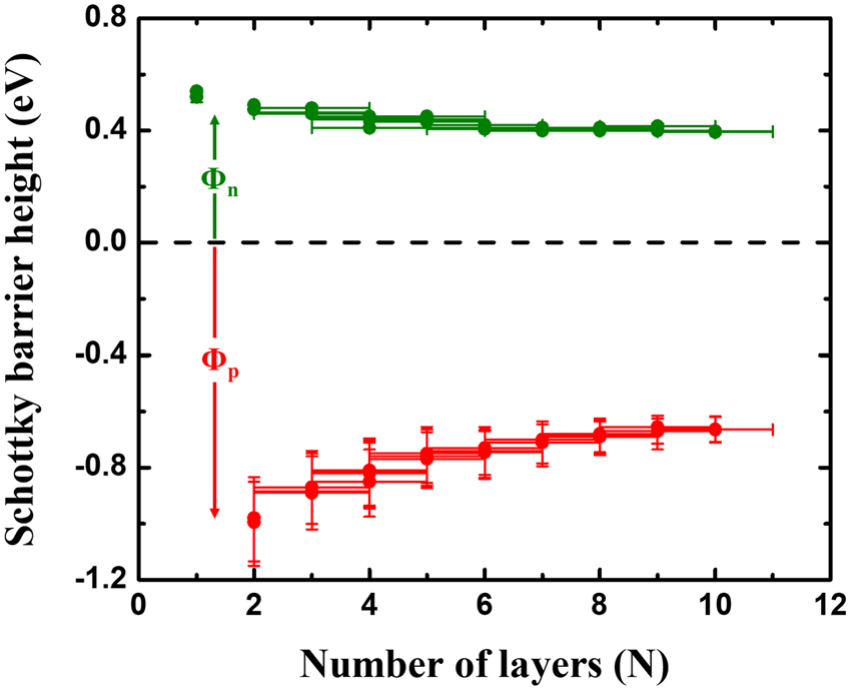
Extracted Schottky barrier heights as a function of flake thickness for WSe_2_ with Ni as the contact metal.

Simulations based on this two-path model match well with the transfer characteristics of all devices for various body thicknesses as shown in Fig. 4. In total more than 28 devices have been fabricated and the characteristics in Fig. 4 are good representations of all devices included in this study. It is important to note that apart from the Schottky barrier heights Φ_n_, only two parameters – the electron effective mass of 0.36m_0_ and the channel thickness dependent dielectric constant - were used as input parameters for the new model and both of those were taken from the literature [references^44,49^ and supplementary information I]. Moreover, the Schottky barrier heights for electrons Φ_n_ obtained using the two-path model (Fig. 5) are in good agreement with previously reported values^32,46^ considering that in these articles the bandgap was assumed to have a certain value. Though the simulated curves shown in Fig. 4 assume a square root scaling length λ_S_ for path-1, employing the generalized scale length λ_T_ does not make a considerable difference, since the contributions of path-1 to the current are negligible in the WSe_2_ FETs as illustrated in Fig. 3(d). Also, for simulations, band movement for path-1 is assumed to slow down beyond its threshold V_TP1_, though the impact of this assumption on the final curve is negligible as mentioned in the previous paragraph.

Deviation of the experimental curves from the simulated ones at high current levels are expected as the transport at such high currents involves a substantial number of injected charge carriers causing scattering in the channel - both underneath the contacts and in the conventional channel. The accumulation of carriers in or close to the device ON-state also implies a reduced gate response that is not captured by our model, which is valid only below threshold. In fact, to describe the ON-state performance of TMD devices a complicated interplay between mobility, carrier density, density of states in the channel, intra and inter-layer resistances and the gate controlled Schottky barriers need to be simultaneously taken into account^54–59^, which is not the topic of this study.

Since the current contribution due to path-2 is proportional to L_contact_ because of operation in the device OFF-state, it can be reduced by decreasing L_contact_. Also, as mentioned before, the band movement for path-2 is much slower than that for path-1 and the relative strength of the gate control depends on the details of the material system and in particular the dielectric constants. Thus, for certain material systems and/or contact lengths the current injection via path-1 can turn out to be considerably higher than that via path-2 and the conventional Schottky barrier model is applicable. An example of this case that is closely related to our previously reported analysis of black phosphorus devices^31^ is discussed in the supplementary information IV. Also, in 1D channels like nanotubes and nanowires one frequently finds device layouts where contacts encase the channel to a large extent and screening prevents the applicability of our model.

Last, we used the above insights into the electron Schottky barrier height Φ_n_ as a function of layer number in combination with our previous findings on the change of transport bandgap E_g_ with body thickness for WSe_2_^37^ to determine the Schottky barrier height for hole injection Φ_p_ using the equation E_g_ = Φ_n_ + Φ_p_. The Schottky barrier heights thus extracted are plotted in Fig. 5 as a function of flake thickness. It is apparent from Fig. 5 that while Φ_n_ changes by only ~100 meV over the thickness range presented, most of the bandgap change occurs in accord with a change of Φ_p_.

## Conclusion

In conclusion, we have proposed a comprehensive, physics-based model to describe the electrical response of back-gated Schottky barrier FETs with an ultrathin body channel by considering an additional current path for the first time. The new model was validated by means of comparison with a sizable amount of electrical characteristics from devices encompassing a wide range of channel thicknesses. Most importantly, in this study we have unveiled the significant role of the channel portion underneath the contacts in describing the carrier transport in transistors with 2D materials employed as channel materials.

### Data availability

The datasets generated during and/or analyzed during the current study are available from the corresponding author on reasonable request.

## Electronic supplementary material

Supplementary Information
