# Supplementary material for: Understanding contact gating in Schottky barrier transistors from 2D channels
Source: Sci Rep. 2017 Oct 3;7:12596. doi: 10.1038/s41598-017-12816-3 (PMC5626721; doi:10.1038/s41598-017-12816-3)
Supplement: Supplementary file 1 — Supplementary Information [file 41598_2017_12816_MOESM1_ESM.pdf]

Understanding contact gating in Schottky barrier transistors from 2D channels

Supplementary Information

Abhijith Prakash[1, 2,]*, Hesameddin Ilatikhameneh[1, 3], Peng Wu[1, 2] and Joerg Appenzeller[1, 2]

[1] School of Electrical and Computer Engineering, Purdue University, West Lafayette, Indiana 47907, USA.

[2] Birck Nanotechnology Center, Purdue University, West Lafayette, Indiana 47907, USA.

[3] Network for Computational Nanotechnology, 207 S. Martin Jischke Drive, West Lafayette, Indiana 47907, USA.

*Address correspondence to prakash1@purdue.edu

I. Body thickness dependent dielectric constants in WSe_2

Similar to other 2D crystals, the dielectric constant, both in-plane (x-direction) and out-of-plane (y-direction), of WSe_2 depends on its thickness. Since the values of dielectric constants for WSe_2 have been reported in [1] only for a few body thicknesses, we have employed a spline interpolation approach to determine the dielectric constants in WSe_2 as a function of body thickness, with the dielectric constant values reported in [1] acting as fixed points. Figure S1(a) and figure S1(b) show the resulting in-plane dielectric constant values ($\varepsilon_{\text{body}-x}$) and out-of-plane dielectric constant values ($\varepsilon_{\text{body}-y}$) employed for the calculations in the main manuscript, respectively.

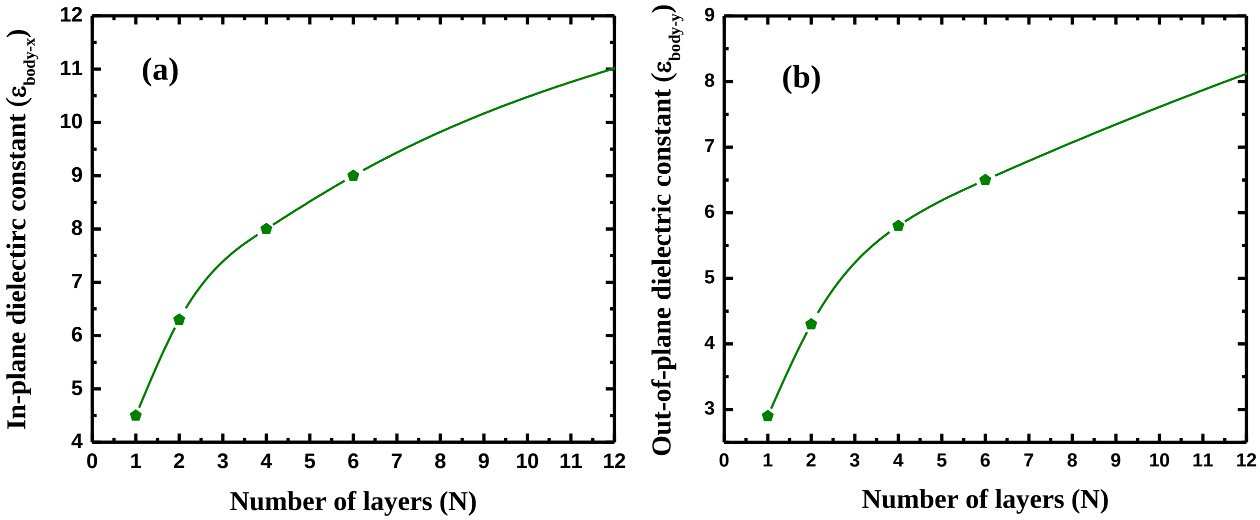

Figure S1: Spline-connected plots of (a) in-plane dielectric constant and (b) out-of-plane dielectric constant in WSe_2 as a function of its body thickness.

II. Ambipolar device characteristics

Figure S2 presents the same set of experimental transfer characteristics as in figure 1(b) in the main text, with both the electron and hole current branches shown.

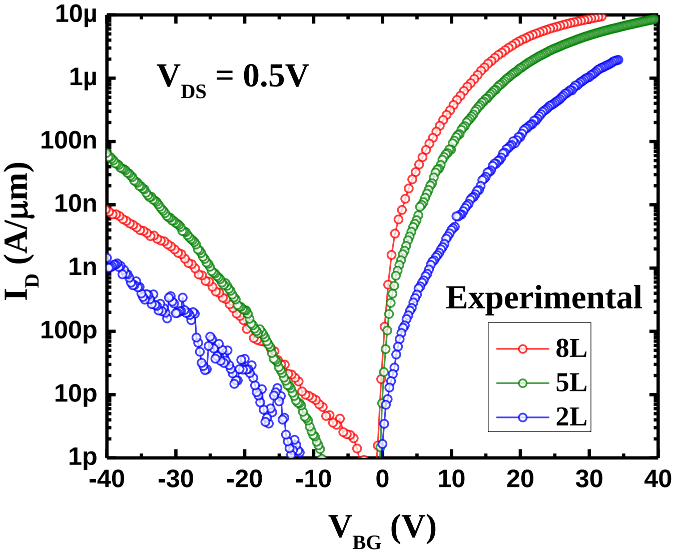

Figure S2: Representative set of ambipolar experimental device characteristics.

III. Current level adjustment using the conventional SB-FET model

An attempt is made in figure S3 to check if artificially selecting a smaller Schottky barrier height, even if it is unrealistic, could help to describe the experimental device characteristics within the conventional SB-FET model. Figure S2 clearly shows that even under these assumptions the full experimental device characteristics are not captured by the model.

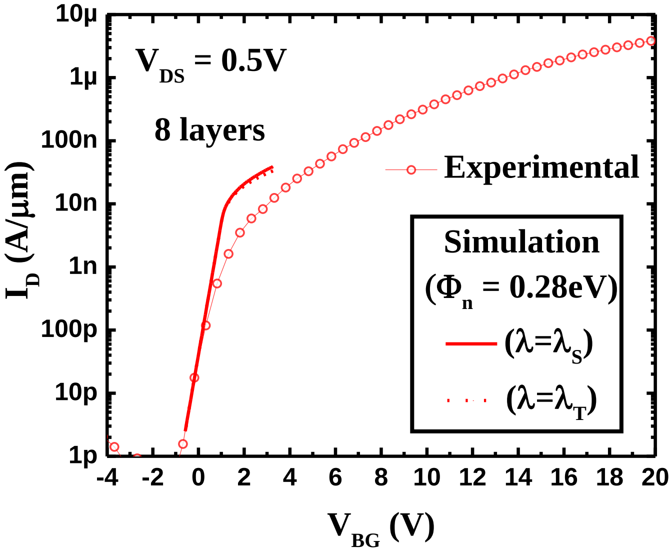

Figure S3: Comparison of the experimental transfer characteristic of an 8 layer WSe_2 device with simulations based on the conventional SB-FET model using an artificially selected Schottky barrier height of 0.28eV.

IV. Illustration of a special case

Presented here is an example of a back-gated SB-FET where currents due to path-2 are less than those due to path-1 for a range of back-gate voltages (V_{BG}). For this example, the Schottky barrier height Φ_n has been chosen to be 0.2eV for a 5nm thick channel. An isotropic dielectric constant of 10 and an effective mass of $0.14m_0$ have been assumed for the channel material for a contact length $L_{contact}$ of 250nm. The resulting simulated electron currents for both paths discussed in the main text are shown in figure S4(a). Since currents predicted by the conventional SB-FET model are much larger than those due to path-2, the conventional SB-FET model can be used to explain the device characteristics fairly well (figure S4(b)). It should be noticed that the material parameters assumed in this example for electron transport match the corresponding parameters for hole transport in Black Phosphorus (BP) if anisotropic transport contributions in BP are ignored [2-5]. Thus, hole transport in BP, as described by Penumatcha, A. V. et. al. [3] is a good example of a 2D channel where the discussed contact gating effects are less pronounced in the resulting device characteristics. (The contact length assumed here is representative of the contact lengths designed in [3]).

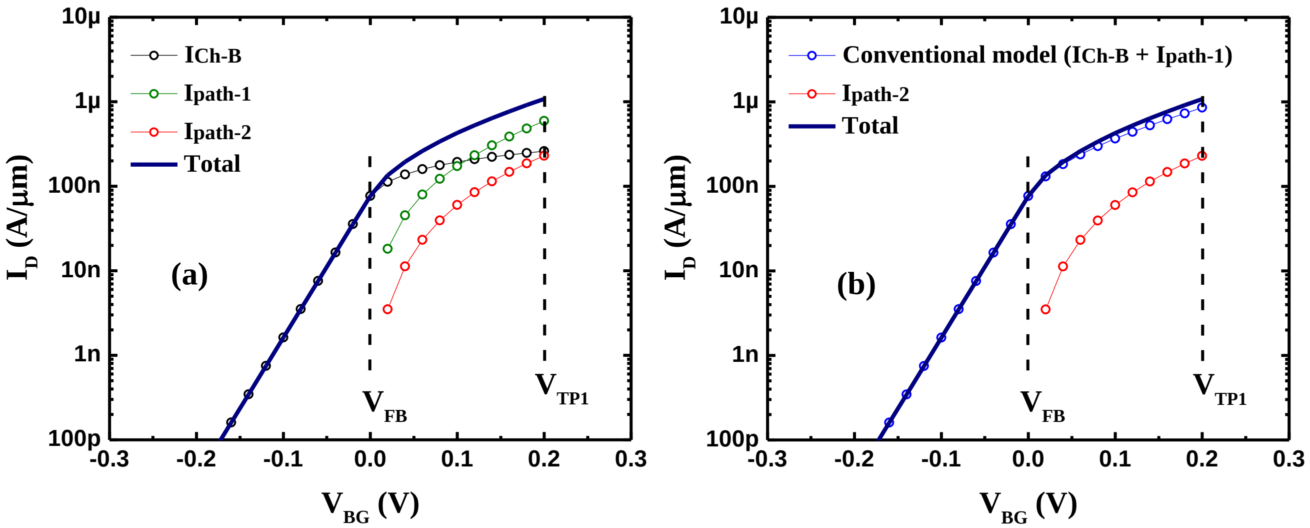

Figure S4: Illustration of a case where currents due to path-2 are significantly less than those predicted by the conventional SB-FET model, such that the conventional SB-FET model can be used to describe the device characteristics.

V. AFM images

Shown below are the AFM images of a few representative WSe_2 flakes used in our study.

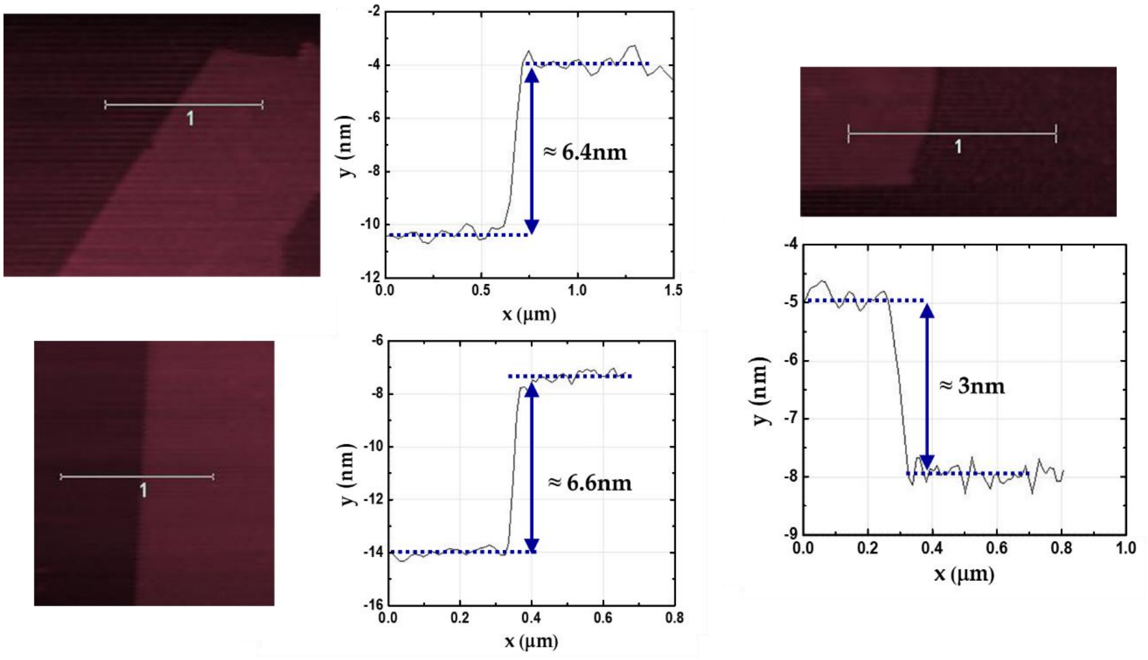

Supplementary note: The validity of $I_{path\text{-}2}$ being proportional to $L_{contact}$ through $N_i(E)$ is limited to the device OFF state, where a finite carrier density underneath the contacts and in the channel can be ignored. In this gate voltage range, the scattering limited current in the device ON state never becomes the dominant resistance of the entire system, in which case the current scaling with the contact length would obviously not be applicable anymore. Moreover, for very large contact lengths, we expect that the linear dependence of $N_i(E)$ on $L_{contact}$ will no longer hold true even in the OFF state, since the access resistance to the channel itself would become dominant, rather than the injection into the TMD.

Supplementary References

1. Kumar, A. & Ahluwalia, P. K. Tunable dielectric response of transition metals dichalcogenides MX_2 (M=Mo, W; X=S, Se, Te): effect of quantum confinement. *Phys. B* **407,** 4627-4634 (2012).
2. Morita, A. Semiconducting black phosphorus. *Appl. Phys. A* **39,** 227– 242 (1986).

3. Penumatcha, A. V., Salazar, R. B. & Appenzeller, J. Analysing black phosphorus transistors using an analytic Schottky barrier MOSFET model. *Nat. Commun.* **6,** 8948 (2015).
4. Ameen, T. A., Ilatikhameneh, H., Klimeck, G. & Rahman, R. Few-layer phosphorene: an ideal 2D material for tunnel transistors. *Sci. Rep.* **6,** 28515 (2016).
5. Ilatikhameneh, H., Ameen, T., Novakovic, B., Tan, Y., Klimeck, G. & Rahman, R. Saving Moore's law down to 1 nm channels with anisotropic effective mass. *Sci. Rep.* **6,** 31501 (2016).
